# Microbial Diversity in Vehicle Windshield Washer Reservoirs: Findings from *Legionella* Screening

**DOI:** 10.3390/microorganisms14010105

**Published:** 2026-01-04

**Authors:** Jaqueline T. Bento, Ana Machado, Adriano A. Bordalo, Eliane Silva, João Rodrigo Mesquita

**Affiliations:** 1 School of Medicine and Biomedical Sciences (ICBAS), University of Porto, 4050-313 Porto, Portugal; jtbento@icbas.up.pt (J.T.B.); ammachado@icbas.up.pt (A.M.); bordalo@icbas.up.pt (A.A.B.); 2 Interdisciplinary Centre of Marine and Environmental Research (CIIMAR), University of Porto, 4450-208 Matosinhos, Portugal; 3 Research Center in Biodiversity and Genetic Resources (CIBIO/InBIO), University of Porto, 4485-661 Vairão, Portugal; 4 BIOPOLIS Program in Genomics, Biodiversity and Land Planning, CIBIO, Campus de Vairão, 4485-661 Vairão, Portugal; 5 Associate Laboratory for Animal and Veterinary Science (AL4AnimalS), 1300-477 Lisboa, Portugal; 6 Centro de Estudos de Ciência Animal (CECA), Instituto de Ciências, Tecnologias e Agroambiente (ICETA), University of Porto, 4051-401 Porto, Portugal

**Keywords:** *Legionella*, windshield, Portugal, microbial diversity

## Abstract

Legionnaires’ disease remains a relevant public health concern, with transmission linked to droplets from diverse aquatic environments, and its burden across Europe, including in Portugal, has been trending up. Vehicle windshield washer reservoirs have been proposed as potential, yet underexplored, habitats for *Legionella* spp. In this study, we investigated 62 windshield washer fluid samples collected in central Portugal. Cultivation on selective BCYE agar supplemented with GVPC and subsequent molecular identification revealed no evidence of *Legionella* spp. However, 23 morphologically distinct bacterial isolates were recovered, and sequencing confirmed diverse taxa from the genera *Brevundimonas*, *Sphingomonas*, *Ralstonia*, and *Xanthobacter*. These findings indicate that washer reservoirs can sustain microbial communities characterized by environmental resilience and biofilm-forming potential, traits that overlap with ecological niches exploited by *Legionella*. Although no *Legionella* was detected, this work represents the first systematic survey of windshield washer reservoirs in Portugal, emphasizing their potential role as overlooked microbial ecosystems and highlighting the importance of continued surveillance. Broader characterization of microbial communities in such artificial aquatic systems may yield insights into microbial interactions that shape pathogen persistence and suppression.

## 1. Introduction

Legionnaires’ disease is a form of atypical pneumonia that can present with clinical features overlapping those of pneumococcal or other bacterial pneumonias [1]. The infection results from inhalation of aerosols contaminated with *Legionella* bacteria, especially *Legionella pneumophila*, which thrives in aquatic environments, including freshwater systems, hot water tanks, cooling towers, and plumbing networks [2]. It can also persist in soils, particularly moist soils rich in organic matter [3]. After inhalation of contaminated aerosols, the bacteria reach the lower respiratory tract, where they replicate intracellularly within alveolar macrophages, evading host immune defenses and triggering an inflammatory response that contributes to lung tissue damage [4,5,6,7].

Over the past two decades, Legionnaires’ disease has become a growing public health concern globally, with increasing incidence reported across Europe, North America, and other regions [8]. According to the European Centre for Disease Prevention and Control (ECDC), more than 10,000 cases are reported annually in Europe, although the real number is believed to be higher due to underdiagnosis and underreporting [9]. This underestimation results partly from diagnostic challenges, since clinical manifestations are non-specific and laboratory testing is not systematically performed in all pneumonia cases. Additionally, environmental surveillance coverage is highly variable across countries, contributing to incomplete understanding of the bacterium’s ecological distribution [10]. The infection often occurs sporadically but may also manifest in outbreaks, which typically arise from anthropogenic water systems providing optimal conditions for bacterial proliferation [11]. Globally, reported outbreaks often involve large numbers of individuals, highlighting the importance of surveillance in public spaces, hospitals, hotels, and industrial facilities [12]. Climate change and aging infrastructure may also contribute to the increased occurrence of outbreaks, as warmer temperatures and stagnation favor bacterial growth. Understanding these factors is key for designing effective prevention strategies and public health policies [13].

In Portugal, recurrent detection of *Legionella pneumophila* (*L. pneumophila*) sequence type 1905, including a major outbreak in 2014 and sporadic cases through 2022, underscores the sustained public health relevance of this strain [14,15]. Since 2014, ST1905 has been repeatedly identified in both clinical and environmental samples across multiple municipalities in the Lisbon region, extending well beyond the original outbreak epicenter [16]. This continued detection suggests the presence of stable environmental reservoirs or recurrent introductions from common sources, reflecting the strain’s persistence and geographical spread. The repeated occurrence of ST1905 in Portugal also underlines the need for continuous public education regarding water system maintenance, as improper disinfection of domestic, industrial, or municipal water systems can increase exposure risk. Integrated approaches combining epidemiological, environmental, and genomic data can help identify high-risk areas and populations [16].

*Legionella* spp. are Gram-negative bacteria ubiquitously found in freshwater environments, persisting as free-living organisms, in close association with protozoa, and in biofilms [4]. Among the more than 60 species described, *L. pneumophila* is the most clinically relevant, accounting for the majority of cases of Legionnaires’ disease and Pontiac fever [17,18], the latter not causing pneumonia. Legionellosis outbreaks are frequently linked to aerosol-producing sources of warm water, such as cooling towers and spas [19]. However, transmission has also been associated with unheated systems, including ornamental fountains, as well as domestic water distribution networks [5,19,20]. Nevertheless, sources of transmission are not always identified, and unusual or poorly understood reservoirs for these pathogens do exist, emphasizing the need to investigate nontraditional aquatic niches [21]. Emerging research suggests that man-made environments outside traditional water systems may serve as potential reservoirs. These may include recreational water features, decorative fountains, and other confined water systems, which can sustain bacterial growth if not properly maintained. Understanding these nontraditional reservoirs is important for designing comprehensive surveillance and mitigation strategies.

Some studies have hypothesized or reported the occurrence of *Legionella* spp. in windshield washer fluids, raising concerns regarding their possible contribution to sporadic cases of Legionnaires’ disease [6,19,22,23]. The mechanism of exposure is biologically plausible, as washer fluid is aerosolized when sprayed onto the windshield, entering the vehicle cockpit, and potentially inhaled by drivers or passengers [22]. Furthermore, occupational groups with frequent vehicle use may be particularly exposed if reservoirs harbor pathogenic bacteria [23]. Additional factors influencing bacterial survival in windshield washer reservoirs may include fluid composition, temperature fluctuations, and frequency of use. Seasonal variations and environmental contamination could also affect bacterial presence. Therefore, these man-made water systems may serve as previously overlooked sources of exposure, especially in regions with high vehicle usage.

Although the epidemiological contribution of windshield washer systems to legionellosis remains uncertain, their potential as overlooked reservoirs warrants systematic investigation. Given Portugal’s temperate climate, extensive vehicle use, and documented *Legionella* activity, it is plausible that such reservoirs may contribute to the local ecology of the bacterium. Investigating these environments may not only clarify potential routes of transmission but also reveal microbial interactions that either suppress or facilitate *Legionella* persistence.

To our knowledge, windshield washer reservoirs have not been investigated in Portugal at all. Considering climatic conditions, widespread vehicle use, and the high incidence of *Legionella* infections documented in the country [16], it is relevant to explore whether such reservoirs may contribute to the local ecology and transmission dynamics of the bacterium. Addressing this knowledge gap may strengthen epidemiological surveillance and advance global understanding of unconventional, artificial environments capable of harboring *Legionella*. Investigating these reservoirs could also inform public health guidance for vehicle maintenance and highlight new interventions to reduce bacterial survival in daily-use water systems.

Understanding the influence of washer fluid chemistry and vehicle operational patterns could help identify modifiable factors that reduce microbial survival, representing actionable mitigation opportunities. In addition, vehicle-associated water reservoirs may host a diverse microbial community. Characterizing this community, a broader understanding of the ecological dynamics within this environment may emerge, eventually revealing microbial interactions relevant to the survival of *Legionella*. Additionally, vehicle-associated reservoirs likely harbor diverse bacterial communities capable of interacting with or outcompeting *Legionella*. Studies of these microbial interactions could reveal ecological constraints or facilitators of pathogen colonization, enhancing our understanding of bacterial survival in chemically selective, man-made environments. Exploring this microbial ecology may reveal mechanisms influencing the establishment of pathogens in chemically selective, man-made environments.

Given the public health significance of *Legionella* and the ubiquity of motor vehicles, this study investigates windshield washer reservoirs as potential habitats for *Legionella* spp., while also providing insights into the bacterial community inhabiting this overlooked aquatic environment. The findings may inform public health policy, risk assessment, and preventive strategies for unconventional reservoirs of *Legionella* in urban and temperate settings.

## 2. Materials and Methods

### 2.1. Sample Collection

In 2024, a total of 62 windshield washer fluid samples were collected from private light-duty vehicles operating in the central region of Portugal. Sampling took place during early summer and included urban and peri-urban areas, ensuring representation of different usage patterns. All samples were obtained directly from the windshield washer reservoirs under sterile conditions, to avoid external contamination. Sample volumes ranged between 2 and 5 mL per vehicle. Although a few reservoirs contained commercial detergent-based washer fluids, this factor did not show any apparent influence on the microbial outcomes observed in the study. Following collection, each sample was kept in an ice chest, until further analysis.

### 2.2. Bacterial Growth Conditions

All fluid samples were analyzed for the presence of *Legionella* spp. From each sample, an aliquot of 0.2 mL was aseptically inoculated, in duplicate, onto Buffered Charcoal Yeast Extract (BCYE) agar containing L-cysteine, supplemented with GVPC (glycine, vancomycin hydrochloride, polymyxin B sulfate, cycloheximide), using the spread plate technique. The procedure was performed in accordance with ISO 11731:2017—Water quality—Enumeration of *Legionella* [24] reference method [18].

Inoculated plates were incubated at 36 ± 1 °C for 10 days in a humidified environment to promote the recovery and growth of *Legionella* colonies. Plates were inspected daily from day 3 onward for colony development and morphological characteristics consistent with *Legionella*. Following incubation, presumptive colonies were screened under UV light to assess the presence of the characteristic bluish-white fluorescence typical of *Legionella* spp. [25]. Colonies that developed on BCYE agar during the 10-day incubation period, exhibiting typical and atypical *Legionella* morphological characteristics, were subjected to molecular identification.

### 2.3. Genomic DNA Extraction

Genomic DNA was extracted from each bacteria isolate using the QIAamp DNA Mini Kit (Qiagen, Hilden, Germany), with automated processing on the QIAcube^®^ platform (Qiagen), following the instructions from the manufacturer. Briefly, from bacterial colonies grown on BCYE agar, a sample suspension was prepared by resuspending a loopful of colonies in 1 mL of sterile water. After, 140 μL of this suspension was used for the genomic DNA extraction. Then, DNA concentrations were quantified with a Qubit 4.0 fluorometer (Thermo Fisher Scientific, Waltham, MA, USA) with the Quant-iT™ 1X dsDNA High Sensitivity (HS) Assay Kit (Thermo Fisher Scientific), following the manufacturer’s instruction, prior to PCR amplification.

### 2.4. PCR Amplification of the Bacterial 16S rRNA Region

Conventional PCR amplification of the full-length 16S rRNA region was performed using primers 27f (5′-AGAGTTTGATCMTGGCTCAG-3′) and 1492R (5′-GGTTACCTTGTTACGACTT-3′) [26]. Reactions were prepared with Xpert AmpliFi Hotstart Mastermix (GRiSP^®^, Porto, Portugal) using 2.0 μL of genomic DNA template according to the instructions from the manufacturer and were run on a Bio-Rad thermocycler (Bio-Rad, Hercules, CA, USA). The cycling program consisted of an initial denaturation at 95 °C for 1 min, followed by 35 cycles of 95 °C for 15 s, 60 °C for 15 s, and 72 °C for 1 min, with a final extension step at 72 °C for 10 min. The amplified products were analyzed on a 1.0% (*w*/*v*) agarose gel stained with Xpert Green DNA Stain direct (GRISP, Porto, Portugal) and visualized under UV-light (Bio-Rad, Hercules, CA, USA). The amplicons (1463 bp) were directly sequenced at STAB Vida (C. Caparica, Portugal) after being purified using the Xpert Exo/SAP 1 tube (GRISP, Porto, Portugal) using the same primers. A negative control (no-template control) was included in each PCR run to monitor possible contamination. For quality assurance, positive controls containing DNA from reference bacterial strains were included in each reaction set to confirm amplification performance and reproducibility.

### 2.5. Sequencing

The resulting bacterial sequences were manually inspected, trimmed, and assembled into consensus sequences using BioEdit Sequence Alignment Editor v7.1.9 software. Consensus sequences were queried against the NCBI GenBank nucleotide database (https://blast.ncbi.nlm.nih.gov/Blast.cgi, accessed on 11 September 2025) for species-level identification based on sequence similarity. Alignments with ≥99% nucleotide identity to reference sequences were considered reliable for taxonomic assignment.

### 2.6. Phylogenetic Analysis

The phylogenetic relationships of the isolates obtained in this study were assessed in comparison with reference sequences from GenBank, along with their respective accession numbers. The phylogenetic analysis was conducted in MEGA version X software, employing the Maximum Likelihood approach with the Kimura 2-parameter model and gamma distributions (K2 + G model) to account for rate variations among sites. Node support was evaluated through 1000 bootstrap replicates. The resulting tree was refined, annotated and visualized using the Interactive Tree of Life (iTOL) platform version 7. All sequences generated in this study were deposited in NCBI GenBank database, and respective accession numbers can be seen in Table 1.

## 3. Results

### 3.1. Bacterial Growth and PCR Amplification of the 16S rRNA Region Analysis

No *Legionella* spp. were isolated from the 62 windshields washer fluid samples analyzed [95% CI: 0.0–5.8%]. Despite culture under selective conditions designed to favor the growth of *Legionella*, all colonies recovered on BCYE (GVPC) medium corresponded to non-*Legionella* bacteria. In total, 23 morphologically distinct bacterial isolates were obtained based on colony appearance, pigmentation, and growth characteristics. The 16S rRNA regions of the 23 morphologically distinct bacterial isolates were successfully amplified by conventional PCR.

### 3.2. Sequencing Analysis

From the 23 amplicons of the 23 morphologically distinct bacterial isolates successfully amplified by conventional PCR, only 17 were successfully sequenced on the full-length 16S rRNA, as the remaining six did not yield usable sequence data. BLASTn sequence analysis of these 17 successfully sequenced bacterial isolates showed a diverse set of bacteria taxa including *Bosea eneae*, *Cupriavidus necator*, *Sphingomonas* sp., *Xanthobacter* sp., *Brevundimonas aurantiaca*, *Ralstonia pickettii*, *Pseudacidovarax intermedius*, *Azorhizobium caulinodans*, *Xanthobacter flavus*, *Brevundimonas vesicularis*, *Caulobacter segnis*, *Roseococcus* sp., *Brevundimonas albigilva and Cytobacillus horneckiae* each with an E-value of 0.0 and percentage of nucleotide identity ranging from 99.31% to 100.00% to reference bacterial isolates (Table 1). All sequences were deposited on the GenBank database under accession numbers PX273813 (isolate 1), PX273856 (isolate 2), PX273896 (isolate 3), PX273897 (isolate 4), PX273898 (isolate 5), PX273899 (isolate 6), PX273900 (isolate 7), PX273906 (isolate 8), PX273907 (isolate 9), PX273909 (isolate 10), PX273910 (isolate 11), PX273911 (isolate 12), PX273915 (isolate 13), PX273916 (isolate 14), PX273930 (isolate 15), PX273931 (isolate 16) and PX273932 (isolate 17).

### 3.3. Phylogenetic Analysis

**Figure 1:**
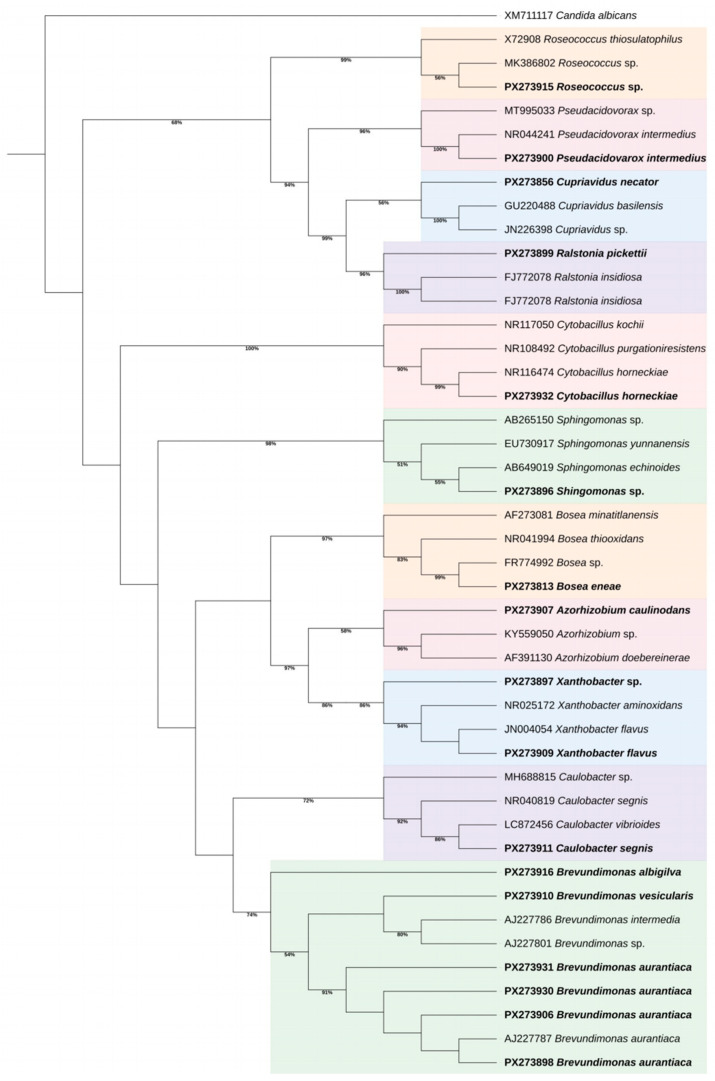
Maximum likelihood phylogenetic tree based on the full 16S rRNA gene region (nucleotide sequences). The phylogenetic relationships of the isolates obtained in this study (highlighted in bold) were analyzed alongside reference sequences retrieved from GenBank, including their respective accession numbers. The analysis was performed using the Maximum Likelihood method with the Kimura 2-parameter + Gamma model (K2 + G) in MEGA X. The percentage of replicate trees in which related taxa are clustered together during the bootstrap test (1000 replicates) is shown next to the branches, with bootstrap values below 50% omitted. The tree is rooted using a Candida albicans strain as outgroup. Colored backgrounds represent distinct genera. The phylogenetic tree was edited using the Interactive Tree of Life (iTOL).

The phylogenetic analysis of the full 16S rRNA gene region showed that all isolates clustered with their corresponding reference taxa in well-supported clades, confirming the accuracy of the molecular identification and illustrating the taxonomic diversity of culturable bacteria inhabiting windshield washer fluid reservoirs (Figure 1). The recovered taxa predominantly belonged to the *Alphaproteobacteria* class, while isolates previously classified as *Betaproteobacteria* are currently assigned to Gammaproteobacteria according to updated genome-based taxonomies. Several of these species are commonly associated with biofilm formation and oligotrophic aquatic systems.

## 4. Discussion

This study represents the first systematic attempt to investigate windshield washer reservoirs in Portugal as potential habitats for *Legionella* spp. Despite the biological plausibility of these systems as reservoirs and previous international reports of *Legionella* detection, no isolates of the pathogen were recovered from the 62 analyzed samples. The investigation of this niche provides a crucial starting point for understanding microbial dynamics in unconventional aquatic systems.

These results also demonstrate the importance of systematically exploring environments that fall outside traditional surveillance frameworks, as unexpected ecological barriers may limit the establishment of pathogens even in seemingly suitable habitats. By documenting these negative findings, this study contributes essential baseline knowledge that will support future comparative assessments across different geographic locations and vehicle-use patterns.

The absence of *Legionella* in the present study may be explained by several factors, including methodological aspects related to the sampling strategy. Specifically, the analysis focused on the fluid rather than on biofilms adhered to reservoir surfaces, where *Legionella* is known to preferentially persist, often intracellularly within protozoa. Moreover, the chemical composition of washer fluids, particularly formulations containing detergents, alcohols, or other additives, may exert inhibitory effects on *Legionella* survival and growth. Under such stress conditions, *Legionella* may enter a viable but non-culturable (VBNC) state, which cannot be detected by culture-based methods, potentially contributing to non-detection despite environmental presence [27]. In addition, environmental stressors such as temperature fluctuations within engine bays, exposure to radiation and intermittent use of reservoirs may reduce bacterial persistence [28]. It is also possible that interactions with other microorganisms present in these reservoirs create competitive or antagonistic dynamics that hinder *Legionella* colonization [29]. Moreover, culture on BCYE (GVPC) detects only culturable *Legionella*; low abundance, stress, or viable but nonculturable cells may evade recovery under these conditions [30]. In addition, the relatively small sample volume analyzed may have contributed to non-detection, as larger volumes (typically 1–2 L) are often required to overcome detection limits in environmental samples. Similar hypotheses have been proposed in previous studies, which reported inconsistent detection of the pathogen in washer fluids across different countries [19,27]. Another important consideration is the potential impact of maintenance practices, such as frequent refilling with commercial fluids or partial drainage during servicing, which may continuously disrupt the establishment of stable microbial populations [31]. Moreover, seasonal variations in fluid composition could create intermittently hostile conditions that further impede Legionella survival [31,32]. Finally, the possibility of transient contamination that escapes detection at single timepoints cannot be excluded, highlighting the value of repeated or longitudinal sampling [33]. Previous studies investigating vehicle-associated water systems and other non-conventional aquatic environments have reported highly variable detection rates of *Legionella*, often linked to methodological and environmental constraints. Moritz et al. (2010) demonstrated that *Legionella* preferentially persists within biofilms and protozoan hosts, which may not be adequately captured through bulk water sampling alone [34]. Similarly, investigations conducted in different geographic and climatic contexts have highlighted the inhibitory role of chemical additives, temperature fluctuations, and intermittent system use on *Legionella* survival [35,36] These studies collectively report inconsistent recovery of *Legionella* from washer fluids and related systems, reinforcing the notion that non-detection in such environments does not necessarily indicate absence, but rather reflects ecological complexity and methodological limitations.

Comparable studies have shown a broad variability in detection outcomes, underscoring the influence of environmental and methodological factors [6,19,27,37]. Such discrepancies have been reported across studies conducted in different geographic and climatic contexts and may reflect differences in environmental conditions, vehicle maintenance habits, or the chemical composition of commercial washer fluids, rather than variability observed within the present study. For instance, ethanol-based formulations may strongly inhibit bacterial survival, whereas detergent-based fluids could permit limited persistence [38]. The diversity of experimental results reported in the literature indicates that the ecology of *Legionella* within artificial water systems cannot be extrapolated from one environment to another [6,19,27,37]. Even within similar reservoir types, factors such as material composition (plastic versus metal tanks for example), storage duration, and frequency of use profoundly influence microbial colonization patterns. Understanding these nuances is essential for interpreting negative findings such as those observed in the present study, which should not be equated with absence of risk but rather contextualized within the limits of detection and environmental variability. This variability also underscores the importance of harmonized protocols for sample processing, culture conditions, and molecular detection to improve comparability across studies. Differences in pre-concentration steps, disinfectant neutralization, or incubation duration may greatly influence detection outcomes, suggesting that future studies would benefit from methodological standardization.

Although *Legionella* was not detected, cultivation on selective medium yielded a diverse range of bacterial taxa, predominantly genera commonly associated with aquatic environments and biofilm formation, such as *Brevundimonas*, *Sphingomonas*, *Ralstonia* and *Xanthobacter* [39,40,41]. Several of these bacteria are known for their metabolic versatility and resilience in low-nutrient conditions, traits that may confer a selective advantage within washer reservoirs [42]. Their presence suggests that these artificial aquatic systems can support stable microbial communities, capable of influencing the ecology of potential pathogens through mechanisms of competition, antagonism, or even facilitation [43,44]. The detection of these genera also indicates that washer reservoirs may represent oligotrophic, selective environments that foster the growth of slow-growing, stress-tolerant microorganisms rather than fast-growing opportunists [45]. These chemically constrained conditions may select for e microbial assemblages that differ from those typically reported in conventional water systems, providing an opportunity to explore microbe–microbe interactions in non-traditional aquatic environments [46].

These findings suggest that windshield washer reservoirs provide chemically selective conditions that support the persistence of structured microbial communities, including genera such as *Sphingomonas* and *Brevundimonas*, which are commonly associated with biofilm formation in engineered water systems [47]. Their coexistence within these systems may alter physicochemical parameters such as pH and nutrient availability, indirectly affecting the viability of *Legionella* or other opportunistic pathogens [48]. The ability of these bacteria to produce extracellular polymeric substances may also support the formation of multi-species biofilms capable of modulating fluid viscosity, nutrient gradients, and surface adherence within reservoirs [46]. Such biofilms may either inhibit pathogen proliferation by competitive exclusion or create micro-niches that could theoretically support VBNC pathogens, highlighting the complex ecological dynamics of these systems [49].

The identification of these environmental bacteria also highlights the relevance of considering microbial interactions in the context of pathogen surveillance. Some species, such as *Sphingomonas* and *Brevundimonas*, are recognized for their ability to form biofilms and interact with protozoa, traits that overlap with ecological niches exploited by *Legionella* [50,51,52]. Thus, while these bacteria are not typically pathogenic to humans, their co-occurrence in shared environments may provide insights into microbial networks that modulate pathogen persistence or suppression [53]. Investigating such interactions may help reveal whether certain bacteria indirectly contribute to the exclusion of Legionella by outcompeting it for resources or by altering environmental conditions. Conversely, some community members might facilitate persistence by producing protective matrices or metabolites, underscoring the need for integrative ecological approaches that consider both pathogens and their accompanying microbiota [53].

From a public health perspective, the absence of *Legionella* in this survey provides reassuring evidence that windshield washer reservoirs may not currently represent a major epidemiological risk in Portugal and may not constitute a major population-level risk in the study setting. The inclusion of vehicle-associated systems in public health discussions may enhance risk communication and improve preventive maintenance practices, particularly in occupational settings. Furthermore, negative findings, when appropriately contextualized, help refine surveillance priorities by identifying environments that warrant more intensive investigation versus those posing minimal risk.

Despite the short sample size, the novelty of this study highlights the importance of continued surveillance to achieve a more comprehensive understanding of the microbial diversity within these reservoirs. Definitive inference is, however, limited by methodological scope and study design, namely, the sample size, single geographic region, and cross-sectional sampling window. Future investigations should address these constraints through longitudinal and season-stratified sampling, and by incorporating direct molecular assays and culture-independent community profiling to capture low-abundance organisms and viable but non-culturable states. Such expanded approaches would also allow the assessment of temporal fluctuations in microbial composition, the impact of weather patterns, and the role of vehicle-use behaviors in shaping microbial communities within washer reservoirs.

Despite the novel nature of this investigation, a few limitations must be acknowledged. The study relied on both cultivation and PCR-based sequencing, which, while informative, may not have captured the full extent of microbial diversity within the samples. The limited volume of washer fluid analyzed may also have constrained the sensitivity of detection. Future studies employing larger-scale sampling combined with metagenomic or other high-throughput molecular approaches could offer a more comprehensive view of the microbial communities inhabiting these reservoirs.

Ultimately, this work expands the current perspective of *Legionella* ecology by considering vehicle-associated environments as part of the urban water continuum. As global mobility and climate conditions evolve, interdisciplinary approaches integrating microbiological surveillance, culture-based and molecular identification methods, and public health oriented risk assessment will be crucial for anticipating emerging risks and refining preventive strategies. The inclusion of mobile, human-associated water systems in ecological research represents an important shift towards understanding pathogen dynamics in increasingly complex built environments. This study lays the groundwork for future interdisciplinary collaborations aimed at identifying under-recognized reservoirs, improving public health preparedness, and adapting surveillance to the rapid environmental and societal changes shaping pathogen ecology.

## 5. Conclusions

This study represents the first systematic investigation of windshield washer reservoirs in Portugal as a potential habitat for *Legionella* spp. Although no *Legionella* was detected among the analyzed samples, the isolation of diverse non-*Legionella* taxa, including genera commonly associated with oligotrophic aquatic environments and biofilms, demonstrates that these reservoirs can sustain microbial communities with ecological traits relevant to pathogen survival and competition. Nevertheless, as this study represents the first systematic investigation of windshield washer reservoirs in Portugal, it highlights the importance of continued monitoring, particularly considering the variability of environmental conditions, and the possible influence of washer fluid formulations on microbial dynamics, unaccounted in this study. Therefore, expanding surveillance efforts will be essential to clarify the potential role of windshield washer reservoirs in the broader ecology of *Legionella*, as well as in the washer liquid concentrated form, and to better understand the microbial networks that may shape pathogen persistence in such artificial environments.

## Figures and Tables

**Table 1:** Bacterial taxa, E-value and highest hits of the 17 morphologically distinct bacterial isolates presented in the windshield washer fluid samples after BLASTn (version 2.16.0 of the software) sequence analysis in the NCBI GenBank database.

Bacterial Isolate	Scientific Name	E-Value	% Identity	Accession Number
1	*Bosea eneae*	0.0	99.31	MN606146
2	*Cupriavidus necator*	0.0	99.93	CP196535
3	*Sphingomonas* sp.	0.0	99.77	KF870453
4	*Xanthobacter* sp.	0.0	99.69	KF560401
5	*Brevundimonas aurantiaca*	0.0	100.00	MN826172
6	*Ralstonia pickettii*	0.0	99.71	HQ696445
7	*Pseudacidovarax intermedius*	0.0	100.00	KX082848
8	*Brevundimonas aurantiaca*	0.0	100.00	NR_028889
9	*Azorhizobium caulinodans*	0.0	99.92	MK014268
10	*Xanthobacter flavus*	0.0	100.00	ON926583
11	*Brevundimonas vesicularis*	0.0	99.92	MN932333
12	*Caulobacter segnis*	0.0	100.00	CP082923
13	*Roseococcus* sp.	0.0	99.54	PP812494
14	*Brevundimonas albigilva*	0.0	100.00	PP087022
15	*Brevundimonas aurantiaca*	0.0	100.00	MN826172
16	*Brevundimonas aurantiaca*	0.0	99.92	MN826172
17	*Cytobacillus horneckiae*	0.0	100.00	CP194732

## Data Availability

The original contributions presented in this study are included in the article. Further inquiries can be directed to the corresponding author.
